# A Modified Recombineering Protocol for the Genetic Manipulation of Gene Clusters in *Aspergillus fumigatus*

**DOI:** 10.1371/journal.pone.0111875

**Published:** 2014-11-05

**Authors:** Laura Alcazar-Fuoli, Timothy Cairns, Jordi F. Lopez, Bozo Zonja, Sandra Pérez, Damià Barceló, Yasuhiro Igarashi, Paul Bowyer, Elaine Bignell

**Affiliations:** 1 Manchester Fungal Infection Group, Institute for Inflammation and Repair, Faculty of Medicine and Human Sciences, Manchester Academic Health Science Centre, The University of Manchester, Manchester, United Kingdom; 2 Department of Environmental Chemistry, Institute of Environmental Assessment and Water Research (IDÆA), Consejo Superior de Investigaciones Científicas, c/Jordi Girona, Barcelona, Spain; 3 Water and Soil Quality Research Group, Institute of Environmental Assessment and Water Research (IDÆA), Consejo Superior de Investigaciones Científicas, c/Jordi Girona, Barcelona, Spain; 4 Catalan Institute of Water Research, ICRA, C/Emili Grahit, 101, edifici H2O Parc Científic i Tecnològic de la Universitat de Girona, Girona, Spain; 5 Biotechnology Research Center, Toyama Prefectural University 5180 Kurokawa, Imizu, Toyama, Japan; Geisel School of Medicine at Dartmouth, United States of America

## Abstract

Genomic analyses of fungal genome structure have revealed the presence of physically-linked groups of genes, termed gene clusters, where collective functionality of encoded gene products serves a common biosynthetic purpose. In multiple fungal pathogens of humans and plants gene clusters have been shown to encode pathways for biosynthesis of secondary metabolites including metabolites required for pathogenicity. In the major mould pathogen of humans *Aspergillus fumigatus*, multiple clusters of co-ordinately upregulated genes were identified as having heightened transcript abundances, relative to laboratory cultured equivalents, during the early stages of murine infection. The aim of this study was to develop and optimise a methodology for manipulation of gene cluster architecture, thereby providing the means to assess their relevance to fungal pathogenicity. To this end we adapted a recombineering methodology which exploits lambda phage-mediated recombination of DNA in bacteria, for the generation of gene cluster deletion cassettes. By exploiting a pre-existing bacterial artificial chromosome (BAC) library of *A. fumigatus* genomic clones we were able to implement single or multiple intra-cluster gene replacement events at both subtelomeric and telomere distal chromosomal locations, in both wild type and highly recombinogenic *A. fumigatus* isolates. We then applied the methodology to address the boundaries of a gene cluster producing a nematocidal secondary metabolite, pseurotin A, and to address the role of this secondary metabolite in insect and mammalian responses to *A. fumigatus* challenge.

## Introduction

In the genomic era of fungal molecular genetics, the context and/or spatial organisation of genes is emerging as an important regulatory determinant [1]. In some instances the mechanistic significance of such organisational structures remains unclear but it is now widely accepted that genes involved in the biosynthesis of certain secondary metabolites are co-localised, in series, as gene clusters [2]. Secondary metabolites (SMs) can be produced by most fungal species [2], [3] and in some cases, such as the biosyntheses of penicillin, sterigmatocystin and aflatoxin by *Aspergillus* species, the genetic regulation of cluster activities has been well characterised [4]–[6]. Many putative SM gene clusters have been inferred by genome sequencing and comparative genomics or by transcriptional analyses where co-regulation of neighbouring genes is in evidence [3], [4], [7]–[9]. Lack of clearly defined biosynthetic pathways for many secondary metabolites means that the boundaries and number of genes comprising each gene cluster are often poorly defined, although common features can be identified including the involvement of polyketide synthases (PKSs) and nonribosomal peptide synthetases (NRPSs), and hybrids thereof [10]. In addition it has been demonstrated that the collective functionality of such gene products is ensured by their chromosomal colocalisation [11], [12]. Noteworthy is the fact that the majority of known and putative SM gene clusters are located at subtelomeric regions of the chromosomes, [8] most likely facilitating their epigenetic regulation by chromatin-based mechanisms [13]. This epigenetic control of secondary metabolism might provide a means by which SM biosynthesis can be tailored to specific growth conditions while remaining otherwise silent.

In the major mould pathogen of humans, *A. fumigatus*, transcriptional upregulation of 70 *A. fumigatus* genes involved in SM biosynthesis was found during initiation of infection in the mammalian lung relative to laboratory cultures [9]. The direct relevance of SM biosynthesis to disease outcomes in whole animals is evidenced by a crucial role for the epipolythiodipiperazine toxin, gliotoxin, in pathogenicity in corticosteroid-treated hosts [14], however, the role of most individual secondary metabolites in pathogenicity of *A. fumigatus* remains a major unanswered question. A clue to the potential relevance of secondary metabolites during mammalian infection is provided by the putative methyltransferase LaeA, which in *Aspergillus* spp. is a major regulator of SM biosynthesis. In *A. fumigatus* a *ΔlaeA* mutant is hypovirulent in mouse models of invasive aspergillosis [15], [16] and transcriptional analysis of a *ΔlaeA* mutant compared to the parental strain showed that LaeA influenced expression of 13 out of 22 secondary metabolite gene clusters [17].

In order to derive functional insight on both gene cluster organisation and the role of the *A. fumigatus* biosynthetic products in fungal pathogenicity, we sought the means to delete and/or re-organise groups of genes. Genetic manipulation of *A. fumigatus* has been fraught with difficulties due to relatively low efficiencies of homologous recombination. Several advances have augmented the success of gene replacements in *A. fumigatus* including the disablement of non-homologous end joining and the exploitation of split-marker strategies to facilitate the direct selection of appropriately mutated transformants [18]–[22]. Specific PCR-based gene targeting strategies have been gainfully employed to achieve deletion of gene clusters in *Ustilago maydis* [23]. In *A. nidulans* the deletion and regulatable expression of gene clusters has been achieved by exploiting highly recombinogenic strains [24].

*Chaveroche et al*. first exploited recombineering for *Aspergillus* gene knockouts by using the large insert sizes of cosmid gDNA clones to maximise homologous integration frequencies in *A. nidulans* [25]. This strategy provided a much-needed solution to the bottleneck then associated with low rates of homologous recombination in *A. fumigatus* and was more widely adopted for deletion of single *A. fumigatus* genes [26] but was limited to DNA insert sizes amenable to cosmid cloning (∼37–52 kb), and reliant upon plasmid-mediated induction of recombinogenic functions. Recent availability of new recombineering reagents, and refinement of culturing and recombineering protocols, has elevated recombineering efficiency and practicability [27]. We have exploited these advances to expand the repertoire of tools available for *A. fumigatus* manipulation. Relative to the previously-used methodology [25], [26] the new reagents promote, via one-step λ-infection of BAC-harbouring *E. coli* clones, a means for higher throughput construction of large recombinant *A. fumigatus* DNA fragments and critically for this study, the ability to work with larger inserts, thereby enabling multiple manipulations of gene cluster architecture from a single BAC clone. A key refinement is the use of a lambda phage which is replication-defective in *E. coli* cells harbouring bacterial artificial chromosomes (BACs), but retains heat-inducible homologous recombination functions. This allows users to render BACs competent for recombineering by a simple lambda infection and to induce recombination in *E. coli* via a simple temperature shift, thereby permitting high throughput manipulations of BAC clones. We used clones from a pre-existing BAC library of *A. fumigatus* genomic clones [28] to delete single genes and gene clusters in *A. fumigatus* by using a modification of this recombineering approach. We standardized the methodology by targeting two, physically unlinked, individual genes: a telomere distal pH-responsive transcription factor-encoding gene *pacC* [18], [29] and a telomere-proximal putative transcription factor-encoding gene *regA*. We then applied the methodology to address the boundaries of a gene cluster producing a nematocidal secondary metabolite, pseurotin A, and to address the role of this secondary metabolite in insect viability and during interactions between *A. fumigatus* and mammalian phagocytic, or respiratory epithelial cells.

## Materials and Methods

### Strains, media and culture conditions

*Aspergillus fumigatus* strains used in this study are presented in Table 1. Fungal strains were routinely grown at 37°C on *Aspergillus* complete medium (ACM) according to Pontecorvo et al. [30] containing 1% (w/v) glucose as carbon source and 5 mM ammonium tartrate as nitrogen source. For solid media 1% (w/v) agar was added. Minimal media (MM) containing 5 mM ammonium tartrate and 1% (w/v) glucose [31] was used for phenotypic testing. For *Aspergillus* transformation MM was supplemented with 1 M sucrose to produce regeneration medium (RM). Liquid cultures were agitated by orbital shaking at 150 rpm unless otherwise stated. For propagation of plasmids, *E. coli* strain XL-10 (Agilent technologies) was grown in Luria-Bertani (LB medium) supplemented with ampicillin (100 µg/ml). The *A. fumigatus* BAC library was maintained in *E. coli* DH10B (Invitrogen, UK). Propagation of BAC clones was performed in LB supplemented with chloramphenicol (12.5 µg/ml). Reagents for recombineering were kindly provided by Donald L Court (Wellcome Trust Sanger Institute, Hinxton, Cambridge, UK). The replication deficient λ phage (λ *cI_857_ ind1* Cro*_TYR26amber_ P_GLN59amber_ rex< >tetra*) [27] was maintained in *E. coli* LE392 (Promega, UK). The BAC library as well as the reagents for recombineering in *A. fumigatus,* are available on request.

**Table 1:** Strains used in this study.

Strain	Genotype	Source
CEA17_Δ*akuB* ^KU80^	CEA17*akuB* ^KU80^:: *pyrG*	[19]
CM237	Wild type	[41]
Af293	Wild type	[47]
PsoA	Af293 *pyr*G^−^ *psoA*:: *pyrG*	[47]
H515	CM237 *paba*A: *hph*	[35]
ATCC46645	Wild type	American Type Culture Collection
Δ*pacC*	CEA17*akuB* ^KU80^:: *pyrG pacC*::BSM-A/H	This study
ΔPsoAcluster	CEA17*akuB* ^KU80^:: *pyrG* PsoAcluster:: BSM-A/H	This study
ΔAFUA_8G00520	CEA17*akuB* ^KU80^:: *pyrG* AFUA_8G00520:: BSM-A/H	This study
ΔAFUA_8G00550	CEA17*akuB* ^KU80^:: *pyrG* AFUA_8G00550:: BSM-A/H	This study
Δ*regA*	CEA17*akuB* ^KU80^:: *pyrG regA*:: BSM-Z/P	This study

*pyrG* encodes an *A. niger* orotidine-5-monophosphate decarboxylase conferring prototrophy to uracil and uridine; BSM-A/H is a biselectable marker constructed during this study which includes the *hph* gene encoding an *E. coli* hygromycin phosphotransferase conferring hygromycin B resistance in *A. fumigatus*; BSM-Z/P is a biselectable marker constructed during this study which includes the *ptr*A gene conferring pyrithiamine resistance in *A. fumigatus*.

### Cloning and BAC recombineering procedures

For standard cloning and sub-cloning procedures the vectors pUC19 [32] or pGEM-T Easy (Promega, UK) were used. All DNA oligonucleotides used in this study were purchased from Sigma (UK) (Table 2). PCRs were performed and optimised according to the manufacturer’s guidelines.

**Table 2:** Oligonucleotides used in this study.

Name	Sequence (5′–>3′)	Purpose in this study
AmpRF	GGTCTGACAGTTACCAATGC	Plasmid construction
Hyg-R	GCTTGATATCGAATTCGTCG	Plasmid construction
Zeo1F	GAATTCTCAGTCCTGCTCCT	Plasmid construction
Zeo1R	CGGGGGATCCACTAGTTCT	Plasmid construction
PtrAF	GGCCAATTGATTACGGGAT	Plasmid construction
PtrAR	ATGGCCTCTTGCATCTTTG	Plasmid construction
pacC BSM-A/H -F	GCGAGCGTCACGTCGGTCG AAAGAGCACAAATAACCTT AACCTGACATGCCAGTGGG GAAGCTGCCGCACCACGACT GTTGGTCTGACAGTTACCAATGC	5′-flanking amplificationand fusion
BSM-A/H -R	TGCTCCTTCAATATCAGTTA ACGTCGACGAGGAAATGTG CGCGGAACCCCTATTTGTTTA	5′-flanking amplification
pacC BSM-A/H -R	TGGATGGAGGGGCGACGCTC TTCGGGGCGAGCACGCTGTAA AGTGCCTCCAGTGTACCGGCG ACGATCATCGTCAAAGATGCTTG ATATCGAATTCGTCG	3′-flanking amplification and fusion
BSM-A/H -F	TAAACAAATAGGGGTTCCGCGCAC ATTTCCTCGTCGACGTTAACTG ATATTGAAGGAGCA	3′-flanking amplification
pacCzeo-F	GCGAGCGTCACGTCGGTCGAA AGAGCACAAATAACCTTAACCT GACATGCCAGTGGGGAAGCTGC CGCACCACGACTGTTTTCTAGAG CGGCCGCGATAT	BSM-Z/P biselectable marker *amplification*
pacCPtrA-R	TGGATGGAGGGGCGACGCTCT TCGGGGCGAGCACGCTGTAAA GTGCCTCCAGTGTACCGGCGA CGATCATCGTCAAAGATGGCC TAGATGGCCTCTTGCA	BSM-Z/P biselectable markeramplification
CF5R	ATAAGGTTAGCCGAGATGCG	*pacC* replacement verification
CF5F	CTAGCACTTCCATGAGCAAC	*pacC* replacement verification
520A-F	CAAAGCCACATCGACCCTT GCCCTCTGGCCGGATCACACCT GGACAAGCTACCCTCCTCTATC GCGTCCTCTTCTTCCTGGGTCT GACAGTTACCAATGC	BSM-A/H biselectable markeramplification
520H-R	GCCGCATCCATATCCAAGCG TGATCTGTAGCTATGTCCCAC TAGGTCTACTCAATGGCATTG TCAGGTCCAGTCCGCCTTGCT TGATATCGAATTCGTCG	BSM-A/H biselectable markeramplification
520-1F	GCCCTCTGGCCGGATCACAC	AFUA_8G00520 deletionverification
520-1R	CATGGGGACTGGCCGCATCC	AFUA_8G00520 deletionverification
550A-F	CGGTCATAGACAAGAGGAATCT CTACATAAAGGCGCTATCCCTGC TATTAAATGACGGGCATGGGATT GGTAGTTCGTAGGGTCTGACA GTTACCAATGC	BSM-A/H biselectable markeramplification
550H-R	CGATTGTACATGCTCACACGTA GAATCGGCACAGTCTTGGGAA AAGTATGTGGTTTAACTAGCC TTGTCGACCTTGCTTTGCTTGA TATCGAATTCGTCG	BSM-A/H biselectable marker amplification
550-1F	CGGGCATGGGATTGGTAGTTCGT	AFUA_8G00550 deletionverification
550-1R	GGCCTAACCGGGTTCCAGCG	AFUA_8G00550 deletionverification
PsoACA-F	AAGGCGGACTGGACCTGACA ATGCCATTGAGTAGACCTAGT GGGACATAGCTACAGATCAC GCTTGGATATGGATGCGGCG GTCTGACAGTTACCAATGC	BSM-A/H biselectable markeramplification
PsoACH-R	TCAGCACTAGGGAAGTCGGT GTAATGGTGTCAGCCTACTCA GTCACGTGCAGGACATAATCC TCCATCCCCCGAACGACAGCTT GATATCGAATTCGTCG	BSM-A/H biselectable markeramplification
PSOAClustF1	CAGCCTGTGGCTCGCTGGTC	*PsoA* cluster deletionverification
PSOAClustR1	TCCCCGCGTCCACACTCGAT	*PsoA* cluster deletionverification
520SB-F	TTCAGGTGCTGCAAGATGTC	Southern Blot probe
520SB-R	CTTCATGGCCGTTCTGGTAT	Southern Blot probe
550SB-F	GGCCTGATCTACCTTCACCA	Southern Blot probe
550SB-R	TAGCAGGGATAGCGCCTTTA	Southern Blot probe
640_F	CCCCTTGACATAGGGTAATAAT GTGCTTTCGCATTGTTCCACCC ATGGCCCCCCCGCGTTCGGAG CTGCGTTAGCTAGGCTTCT AGAGCGGCCGCGATAT	BSM-Z/P biselectable markeramplification
640_R	TTATTTGCTGGCATCTCGCAA CTTCTCAAGAAGATGGACCAA GTTATCCACCAGTGGCGGAGT CTGTTTAGAAGTTTCATATGGCC TCTTGCATCTTTG	BSM-Z/P biselectable markeramplification
640_INT_F	ATTCGGCTCTGCATATCACC	*regA* disruption verification
640_INT_R	TGAATGATAGGCGTCCTTCC	*regA* disruption verification
5′_640	TCCAGGATCTTCGCATAGGT	*regA* disruption verification
3′_640	GACCGAGTTGACTCGGATCT	*regA* disruption verification

Plasmids expressing biselectable markers BSM-Z/P and BSM-A/H respectively conferring Zeocin (Z) and pyrithiamine (P), or ampicillin (A) and hygromycin (H) resistances were constructed as follows. To construct pBSM-Z/P a gene conferring resistance to zeomycin was amplified by PCR from the plasmid pCDA21 [25] using the primers Zeo1F and Zeo1R (Table 2). The amplicon was blunt ended and cloned into the SmaI site of pUC19 to produce the plasmid pZ3. The *ptr*A gene was obtained by PCR amplification from plasmid pPTRII [33] using primers PtrAF and PtrAR (Table 2) and was cloned into the SalI site of pZ3. pBSM-A/H was constructed by PCR amplification of a gene conferring ampicillin resistance from the plasmid pSK379 [34] using primers AmpR-F and AmpRHyg-R (Table 2); and PCR amplification of a gene (*hph*) conferring hygromycin resistance from pID621 [35] using primers AmpRHyg-F and Hyg-R (Table 2). Fusion of the two amplicons was performed by an overlapping PCR procedure, using PrimeSTAR DNA polymerase enzyme (Clontech, UK) and the primers AmpR-F and Hyg-R (Table 2). The resulting PCR product was cloned into the pGEM-T Easy vector (promega, UK) according to the manufacturer’s guidelines.

### Construction of recombinant BACs by recombineering

**Figure 1:**
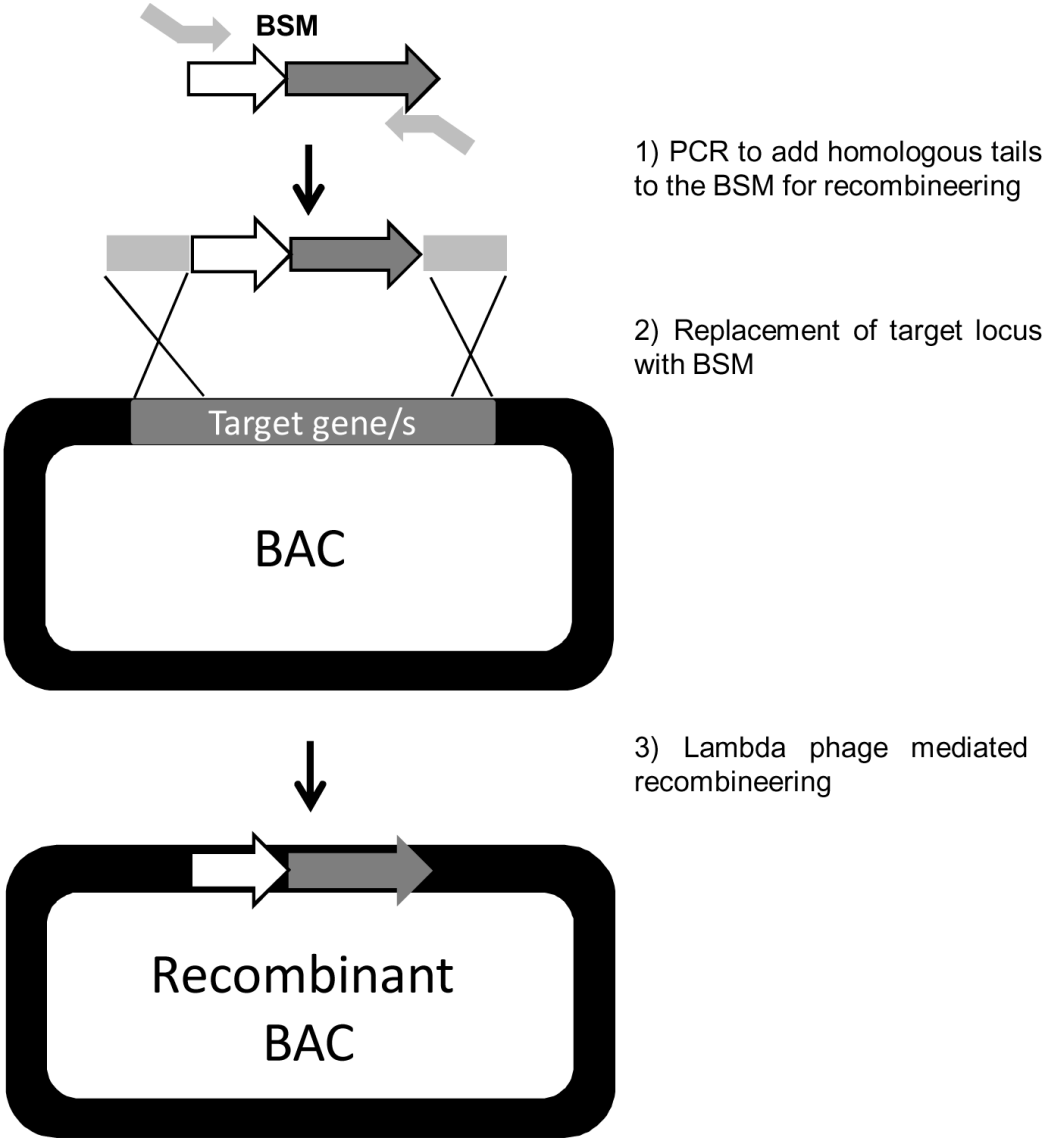
Overview of BAC recombineering in *E. coli*. BSMs were amplified by PCR using tailed oligonucleotide primers: 1) Primer tail sequences were designed to introduce 80 bp of homology to the target locus, at both of the 5′ and 3′ extremities of the BSM. 2) Replacement of target locus with BSM. 3) Heat-induction of homologous recombination functions mediate by lambda phage in *E. coli*.

For construction of recombinant BAC clones a library of end-sequenced, indexed, Af293 *A. fumigatus* BAC clones generated at the Wellcome Trust Sanger Institute (in collaboration with the University of Manchester) [28] was utilised (Table S1). In order to construct gene replacement cassettes for recombineering, biselectable markers (BSMs) were amplified by PCR using tailed oligonucleotide primers. To avoid contamination by BSM-Z/P or BSM-H/P the plasmids were linearized prior to PCR and absence of circular DNA was confirmed by *E. coli* transformation. Primer tail sequences were designed to introduce 80 bp of homology to the target genetic locus, at both of the 5′ and 3′ extremities of the BSM (Figure 1 and Table 2). PCR amplicons for recombineering were generated using the high fidelity DNA polymerase PrimeSTAR (Clontech, UK). Amplicons were purified gel extracted using the purification kit NucleoSpin for gel extraction (Macherey-Nagel, Germany). To generate recombinant BACs, 1 ng of the appropriately tailed PCR amplicon was used as a template for PCR. PCR products were precipitated with 100 µl ethanol and 2 µl 5 M NaCl per 50 µl of PCR reaction. Air-dried PCR products (∼3 µg) were dissolved in 100 mM CaCl_2_ and stored at 4°C until needed.

BACs containing the *A. fumigatus* DNA sequences of interest were selected from the library (Table S1) and recombinant BACs were generated by transformation and heat induction according to the method described by Chan et al. [27], [36]. A precise protocol for this procedure is provided as Protocol S1 in supporting information.

After recombineering BAC DNA extraction was performed with Qiagen reagents for plasmid isolation [36] and verification of insertion or deletion in targeted BACs was obtained by PCR using appropriate primers (Table 2).

### Genetic manipulation of *A. fumigatus*

For fungal transformation BAC DNA was extracted from 50 ml LB cultures. DNA was resuspended in 200 µl of water and 60 µl of recombined BACs (∼1 µg) were linearized overnight with the appropriate restriction enzyme in a total volume of 70 µl. Restriction enzymes were heat inactivated before *A. fumigatus* transformation. Only freshly prepared BAC DNA was used for transformation.

Protoplast transformation was based on the protocol described by Szewczyk and co-workers [37]. Selection of transformants was performed in RM media containing 150 ug/ml of hygromycin or 0.5 ug/ml of pyrithiamine. Plates were incubated at room temperature for 24 hours and then incubated at 37°C for 72–144 hours. Gene targeting was first verified by PCR using primers targeting the corresponding gene, selected outside of the flanking regions and/or in combination with primers targeting the resistance gene marker. For PCR verification DNA was extracted from spores [38]. Single homologous recombination into the *A. fumigatus* genome was confirmed by Southern blot analysis [39] using digoxigenin-labeled probes and the DIG system (Roche, UK) for hybridization and detection.

### *Aspergillus* phenotypic analysis

*Aspergillus fumigatus* strains were evaluated by spotting 4 sequential 10 fold dilutions of candidate *A. fumigatus* spores, starting at a concentration of 2.5×10^4^ per spot, onto MM pH 6.5. Alkaline tolerance was assessed by spotting 2.5×10^4^ spores onto MM pH 8. Plates were incubated at 37°C for 48 hours. Images were captured using a Nikon Coolpix 990 digital camera.

### Quantification of pseurotin A in fungal culture filtrates

Fungal strains were grown at 37°C on *Aspergillus* complete agar (ACM) for 4 days prior to conidial harvest. 200 ml ACM liquid media was inoculated with *A. fumigatus* strains at a concentration of 10^6^ spores per ml. Cultures were incubated at 30°C for 1 week with agitation at 180 rpm. After the incubation period, chloroform (180 ml) was added to each bottle, and the contents were mixed on a rotary shaker at 180 rpm for 15 minutes. After filtration through Miracloth (Calbiochem, USA), the chloroform phase was separated and evaporated to dryness on a rotary evaporator at 40°C. The residue was dissolved in chloroform and filtered. The extracts were evaporated under nitrogen, then reconstituted in 1 ml of methanol and filtered through a 0.22 µm filter (Millipore, UK). The extracts were analyzed for pseurotin A levels using ultra high pressure liquid chromatography coupled to a triple quadruple mass spectrometry (UPLC-(ESI)-QqQ-MS), in positive mode. Pseurotin A (CAS 58523-30-1) purchased from ENZOlife sciences was used as standard while 2-fluoro and 3-fluoro-pseurotin A, obtained in high purity by directed biosynthesis by the group of Dr. Igarashi were used as internal standards [40]. The chromatographic separation of the compounds was performed on a Waters Acquity UPLC BEH C_18_ column (50 mm×2.1 mm, 1.7 um) (Waters, Milford, USA) preceeded by a pre-column of the same packing material (5 mm×2.1 mm, 1.7 um). The mobile phases employed were: (A) acetonitrile and (B) water (20 mM Ammonium Acetate). Elution was accomplished with the following solvent gradient: 0 min (20% A) – 1 min (20% A) – 6 min (30% A), – 6.5 min (95% A) – 6.8 min (20% A) and stabilizing until 8 min. The flow rate was 300 µL min^−1^ and the column temperature was held at 35°C. The injection volume was 10 µL.

### Macrophage phagocytosis assays

Phagocytosis of *A. fumigatus* spores was measured in the murine macrophage cell line RAW 264.7. Macrophages were grown in RPMI media supplemented with 10% fetal bovine serum (containing L-glutamine (200 mM), penicillin (10,000 units/ml), streptomycin (10 mg/ml), Sigma, UK) at 37°C in an atmosphere of 5% CO_2_. Prior to infection, macrophages were adjusted to a cell density of 5×10^5^ cells per ml. 1 ml of cells was added to 24 well Multiwell (BD Falcon) plates and allowed to adhere overnight at 37°C in an atmosphere of 5% CO_2_. Macrophages were challenged with *A. fumigatus* spores at an effector to target (E:T) ratio of 1∶1 and incubated for 2 hours at 37°C in an atmosphere of 5% CO_2_. Next, the residual spores in the culture medium were recovered, washed three times in PBS and colony forming units (CFUs) were enumerated and expressed as a percentage of infectious dose to reflect the proportion of internalised spores.

### Quantification of lactate dehydrogenase (LDH) release from A549 lung epithelial cells

LDH release from A549 monolayers was quantified following co-incubation with *A. fumigatus* conidia at an E:T ratio of 1∶0.1. LDH release was determined using the CytoTox 96 Non-Radioactive Cytotoxicity Assay (Promega). A549 cells were cultured at 5×10^5^ cells/well in 24-well plates for 24 hours in MEM media supplemented with 10% fetal bovine serum and containing L-glutamine (200 mM), penicillin (10,000 units/ml), streptomycin (10 mg/ml) (Sigma, UK) at 37°C in an atmosphere of 5% CO_2_. The assays were performed according to the manufacturer’s instructions and the measurements from three biological replications were evaluated.

### Galleria mellonella survival assay

Wax-moth larvae were infected with *A. fumigatus* CEA17_Δ*akuB* ^KU80^ or transformants (Table 1). CM237 parental [41] and a para-aminobenzoic acid (PABA) auxotroph of CM237 (referred to as *A. fumigatus* H515) [35] were used as positive controls for attenuated and non-attenuated virulence respectively. Wax moth larvae killing assays were carried out as described previously [42], [43]. Briefly, groups of 10 larvae (0.3–0.5 grams, R.J. Mous Livebait, The Netherlands) were inoculated into the haemocoel with 10 µL of a 10^7^ conidia/ml suspension in water for injection so the final inoculum in each group was 10^5^ conidia per larva. Additionally, non-infected larvae, injected with 10 µl of water were included in parallel in every infection. 4 µg/ml of para-aminobenzoic acid (PABA) was used to inject *G. mellonela* infected with the H515 strain. Mortality, defined by lack of movement in response to stimulation and discoloration (melanization) of the cuticle, was recorded daily.

### Statistical analyses

Statistical analyses were performed in GraphPad Prism, version 5. The statistical significance of variances between phagocytosis and cell cytotoxicity was calculated by using a nonparametric Mann-Whitney t test. A p value<0.05 was considered significant. Kaplan-Meier survival curves were analysed by using a log-rank (Mantel-Cox) test for significance. A p value<0.01 was considered significant.

## Results and Discussion

### Optimisation of BAC-mediated gene replacement in *A. fumigatus*

In order to commence a functional genomic analysis of gene clusters and virulence in the human fungal pathogen *A. fumigatus* we developed the means to manipulate gene content of complex genetic loci using BAC-mediated recombineering. A recombineering approach was previously successfully applied in *A. nidulans* [25] where recombinant cosmids were generated in *E. coli* following transformation with a plasmid carrying the λ phage *red*γαβ operon. In our study a higher throughput approach was adopted whereby recombineering functions are transiently supplied, via phage infection and a simple temperature shift, to BAC-harbouring *E. coli* cells [27]. In this manner BAC-cloned genomic regions of interest are replaced with a biselectable marker (BSM) which confers selectable tolerance to antibiotics and/or toxic metabolites in both *E. coli* and *A. fumigatus.* We used a previously constructed library of end-sequenced, indexed *A. fumigatus* BAC clones (Table S1) to facilitate our analysis [28]. This *A. fumigatus* BAC library provides 10X genome coverage and contains 8380 clones having an average insert size of 75 Kb (Table S1 and File S1).

**Figure 2:**
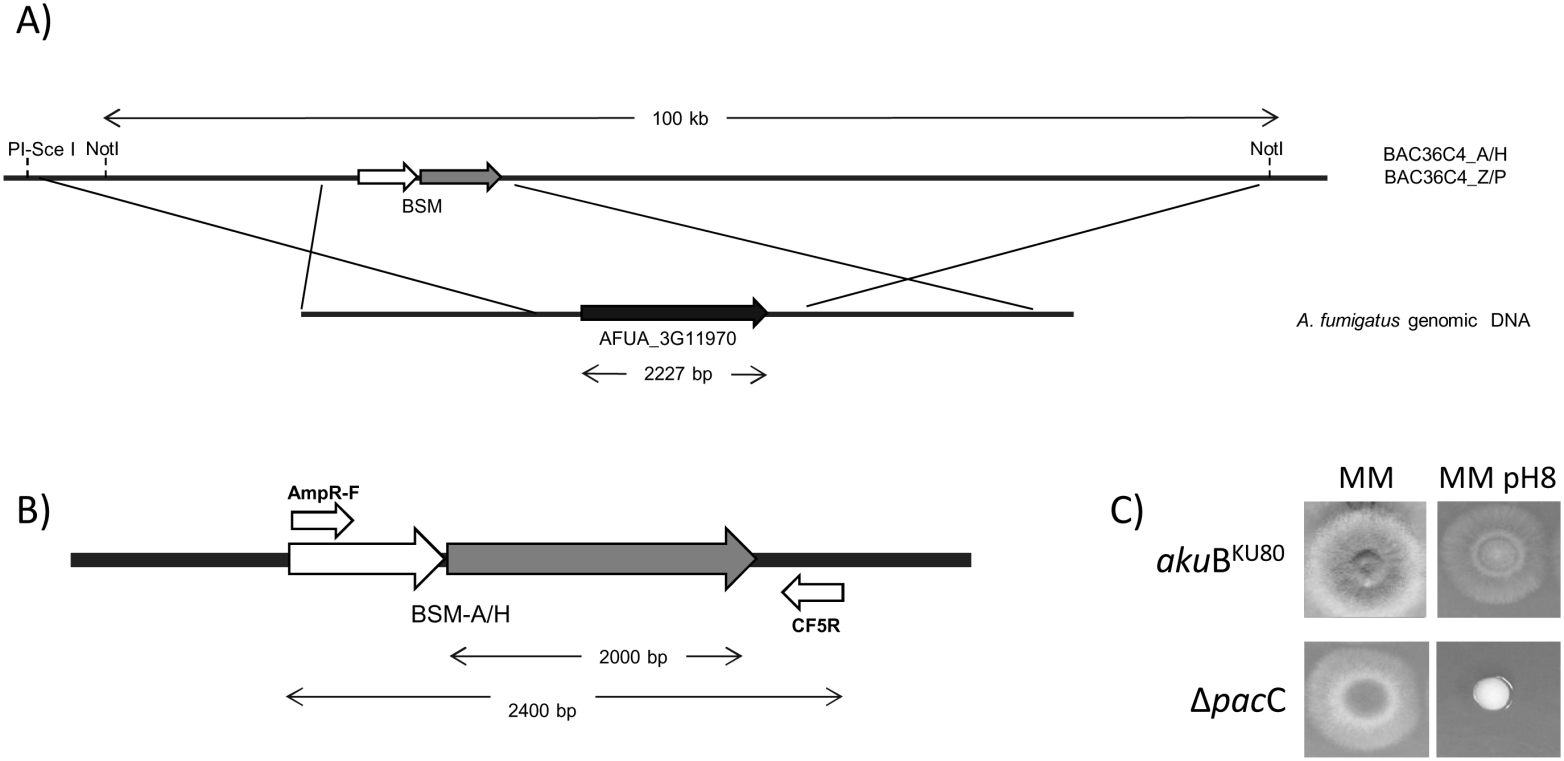
Deletion of the *pac*C gene in *A. fumigatus* CEA17_Δ*akuB* ^KU80^ (referred as *akuB* ^KU80^). A) Schematic view of *pac*C gene deletion. B) Primers used to check gene replacement at the *pac*C locus by PCR. C) Phenotypic analysis of *Δpac*C mutants compared with the wild type. 2.5×10^4^ spores were point inoculated onto MM pH 6.5 and MM pH 8. Plates were incubated at 37°C for 48 hours.

In order to establish and optimise the methodology we first elected to replace single genes, selecting two, physically unlinked, individual genes AFUA_1G17640 and AFUA_3G11970. AFUA_3G11970 is a telomere distal gene encoding the transcription factor, PacC which is involved in alkaline signal transduction [18]. AFUA_1G17640 is a telomere-proximal gene encoding a putative transcription factor, RegA, which resides in a cluster of genes upregulated during murine infection and has a possible role in melanin biosynthesis [44], [45]. For our initial experiments we exploited the well-characterised alkaline sensitivity of PacC null mutants to permit rapid assessment of homologous gene replacements amongst transformants, and utilised two newly constructed biselectable marker plasmids (pBSM-Z/P and pBSM-A/H) to permit comparative assessment of achievable gene replacement frequencies. Schematic overviews of recombinant BAC construction in *E. coli* and BAC-mediated *pacC* gene deletion are provided in Figures 1 and 2A, respectively. A BAC clone (AfB28-mq1_36C04) having appropriate coverage of the *pacC* AFUA_3G11970 genomic locus was retrieved from the library (Table S1). The BAC insert spanned the entire AFUA_3G11970 gene incorporating 24 kb and 74 kb of 5′ and 3′ flanking regions respectively. Appropriately recombined BAC clones were identified by PCR with primers AmpR-F and CF5R (Figure 2B) or PtR-F and CF5R. Recombinant BACs were denoted as BAC36C4-Z/P and BAC36C4-A/H where the AFUA_3G11970 gene had been replaced with an ampicillin/hygromycin or zeocin/pyrithiamine biselectable marker respectively.

In order to linearize the recombinant BACs prior to *A. fumigatus* transformation two methods were used. For transformations using BAC36C4-A/H two different approaches were tested; (i) NotI digestion, which cuts twice in the polylinker of the pBACe3.6 vector and also excises a linear DNA fragment of 100 kb containing 24 kb and 74 kb of *pacC-*flanking 5′ and 3′ sequences respectively, and (ii) PI-SceI digestion which simply linearises the recombinant BAC clone. NotI digestion was not an option prior to transformation with BAC36C4-Z/P, because the zeocin resistance cassette contains a NotI restriction site. Transformants were analysed by PCR using DNA extracted from spores, and primers indicated in Figure 2B and Table 2. First, loss of the *pacC* gene was determined using primers CF5F and CF5R (data not shown). Appropriate insertion of the BSM was subsequently identified with primers AmpR-F and CF5R (Figure 2B) or PtR-F and CF5R. Transformants which had taken up the exogenous DNA were screened for growth at alkaline pH (Figure 2C) to determine the frequency of gene replacement amongst transformants analysed. Table 3 shows the efficiency of allelic replacement at the *pacC* locus when recombinant BACs are linearised according to these two strategies. Regardless of the strategy undertaken, and independent of the BSM utilised, we reproducibly obtained a minimum of 19% of total transformants having undergone gene replacements. We found gene replacements utilising the BSM-A/H biselectable marker to be most favourable due to superior efficiencies of homologous recombination and an easier restriction digestion strategy.

**Table 3:** Efficiency of allelic replacement at the AFUA_3G11970 locus when vectors were linearized with different enzymes.

DNA	Enzyme	Frequency (%) of gene replacement at the AFUA_3G11970 locus/total of transformants
BAC36C4-A/H	NotI	7/17 (41%)
BAC36C4-A/H	PI-SceI	2/6 (33%)
BAC36C4-Z/P	PI-SceI	4/21 (19%)

Keller et al identified that telomere position effect (TPE) can influence the expression of selectable markers targeted to telomeric loci [46]. In order to assess the impact of such effects upon our methodology, and to verify that telomeric loci are amenable to BAC-mediated gene replacements, we constructed a recombinant BAC to replace the *reg*A (AFUA_1G17640) gene, which is positioned 80 kb from the right terminus of Chromosome 1, according to the genome of the sequenced isolate Af293. A BAC clone (AfB28_mq1_17e12) whose insert spanned the entire AFUA_1G17640 gene with 12326 and 1442 bp of 5 and 3 flanking regions respectively, was selected as the recombineering substrate. The zeocin and pyrithiamine biselectable marker was amplified from the pBSM-Z/P plasmid with primers 640_F and 640_R (Table 2) and recombinant BACs were sourced via diagnostic PCR (Figure S1). The recombinant BAC was digested with SacI, liberating a 12.5 kb deletion cassette (Figure S1). SacI digests were heat inactivated and used for subsequent *A. fumigatus* transformations. Homologous integrants were identified by PCR (Figure S1), exploiting the presence of a NotI site in the BSM. *reg*A deletion was verified by Southern blot, probing with a 600 bp fragment of the zeocin cassette, which produced a single fragment of the expected size (Figure S1).

### BAC mediated deletion of the entire pseurotin biosynthetic gene cluster, or associated genes

Pseurotin A is a cyclic peptide putatively biosynthesised by a cluster of five genes housed on the left subtelomeric arm of chromosome 8. Genes in the cluster encode two putative hydrolases (AFUA_8G00530, AFUA_8G00570) a putative methyltransferase (AFUA_8G00550), a putative P450 monooxygenase (AFUA_8G00560) and the hybrid PKS/NRPS *psoA* (AFUA_8G00540) [47]. It has been demonstrated, via gene replacement analyses that integrity of the *psoA* gene is required for the biosynthesis of pseurotin A in *A. fumigatus* [47]. Pseurotin A is a compound that has been reported as a competitive inhibitor of chitin synthase, inducer of nerve-cell differentiation [48] and a suppressor of immunoglobulin E production [49]. Additionally, recent transcriptional, proteomic and metabolic analyses have demonstrated pseurotin A biosynthesis in hypoxic, but not normoxic culture [50] suggesting the production of a toxic and/or immunomodulatory secondary metabolite in hypoxic microenvironments encountered during pulmonary infection [51], [52]. To further understand the regulation of pseurotin A production we constructed *A. fumigatus* mutants lacking the entire pseurotin gene cluster (AFUA_8G00530 – AFUA_8G00580) or other genes within or surrounding the cluster limits. We focused upon AFUA_8G00520, which encodes an integral membrane protein which lies beyond the cluster boundaries but is co-regulated, during murine infection [9], with the genes within the cluster. We also studied the AFUA_8G00550 gene, which encodes a SirN-like methyltransferase predicted to transform an intermediate of the pseurotin A biosynthetic pathway [47], [53].

The pseurotin gene cluster is located 115 kb from the left arm of chromosome 8. Gene sequences conforming to those included within the PsoA gene cluster [47] matched with three different clones from the *A. fumigatus* BAC library (AfB46-09f02, AfB46-09a06 and AfB46-09b06 as indicated in Table S1, and abbreviated in this study to BAC09f02, BAC09a06 and BAC09b06, respectively). Regions within these BAC clones were targeted by recombineering with BSM-A/H^,^ and used for *A. fumigatus* transformation. We worked in parallel with all three clones to demonstrate the versatility of our BAC-mediated approach. Thus, the BAC09f02 clone was utilised to delete AFUA_8G00520, encoding an integral membrane protein. The BAC09a06 clone was utilised to delete AFUA_8G00550, encoding a methyltransferase, and the BAC09b06 clone was utilised to delete the entire cluster of genes (AFUA_8G00530 – AFUA_8G00580). BAC AfB46-09f02 (insert size 56305 bp) contained sequence spanning genes AFUA_8G00420 to AFUA_8G00590 of the Af293 *A. fumigatus* genome, BAC09a06 (insert size 98501 bp) contained sequence spanning genes AFUA_8G00360 to AFUA_8G00740 and BAC09b06 (insert size 119804 bp) contained sequence spanning genes (AFUA_8G00490 to AFUA_8G00900).

**Figure 3:**
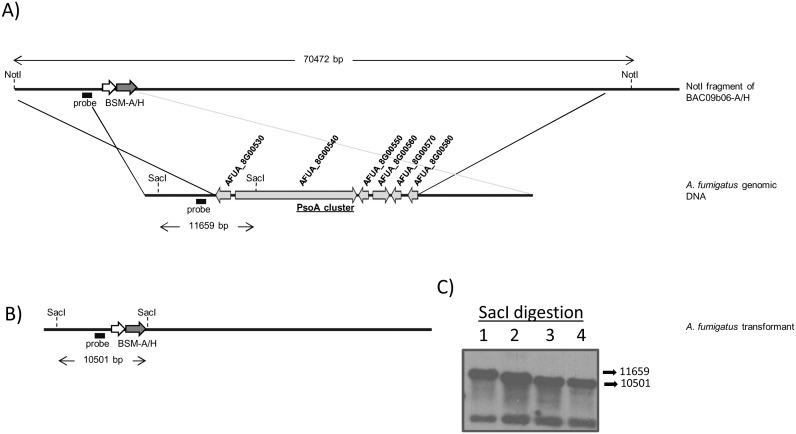
Deletion of the PsoA cluster in *A. fumigatus* CEA17_Δ*akuB* ^KU80^. A) Schematic representation of PsoA cluster replacement by BSM-A/H cassette in *A. fumigatus* CEA17_Δ*akuB* ^KU80^. B) Expected structure of the replacement locus and C) Southern blot analysis of PsoAcluster deleted mutant and wild type (WT) strains. Expected hybridization band pattern: (1) 11659 bp for WT, and (2, 3, 4) 10501 bp for ΔPsoAcluster mutants.

Allelic replacements were first tested by PCR and single integration was confirmed by Southern blot (Figure 3, S2 and S3). Based on the PCR results frequency of homologous recombination in the *A. fumigatus* CEA17_Δ*akuB* ^KU80^ genetic background was high with more than 85% of tested transformants (n = 6–9) undergoing allelic replacement at the correct genomic locus. Although lower frequencies of gene and gene cluster deletions were obtained when the clinical isolate ATCC46645 was used (Table 4), we obtained relevant mutants within the first 8 transformants tested, indicating practically useful, if not heightened, frequencies of gene replacement in non-mutated clinical isolates.

**Table 4:** Efficiency of allelic replacement at the pseurotin A locus using different *A. fumigatus* strains.

A.fumigatus strain	Percentage of appropriate recombinant transformants/total transformants tested by PCR		
	ΔAFUA_8G00520	ΔAFUA_8G00550	ΔPsoAcluster
CEA17_ΔakuB^KU80^	8/9 (88%)	6/6 (100%)	6/7 (85%)
ATCC46645	2/63 (3.1%)	1/8 (12.5%)	1/8 (12.5%)

Our data demonstrate the utility of this method for deletion of single genes, gene clusters and neighbouring genes. Although outside the objectives of this study, our method would also facilitate analysis of specific protein domains of *A. fumigatus* PKS and NRPSs. For example, the hybrid PKS/NRPS gene *psoA* is 12024 bp in length, and encodes a protein having multiple functional domains including those conferring putative acyltransferase, dehydratase, methyltransferase, ketoreductase, acyl carrier, thiolation and reductase activities [47]. Phage based recombineering of BAC09b06, which spans the entire *psoA* gene, could therefore facilitate the rapid deletion of DNA regions which encode distinct activities. This type of targeted mutational approach was used by Hahn and Stachelhaus to generate multiple mutations in the C-terminus of the prokaryotic PKS TycA in order to demonstrate the presence of short communication-mediating (COM) domains [54].

An important caveat to consider with this method is that the BAC clones from the *A. fumigatus* library are derived from the Af293 strain, so genetic replacements in other genetic backgrounds will carry any sequence polymorphisms from the original strain. In fact, comparative genomic analysis of A1163 and Af293 *A. fumigatus* isolates identified the number of unique genes in each genome as up to 2% of total genomic cohorts [55]. It is therefore important, when working in alternative genetic backgrounds to scrutinise/moderate the region of replaced sequence to mitigate the introduction of polymorphisms.

**Figure 4:**
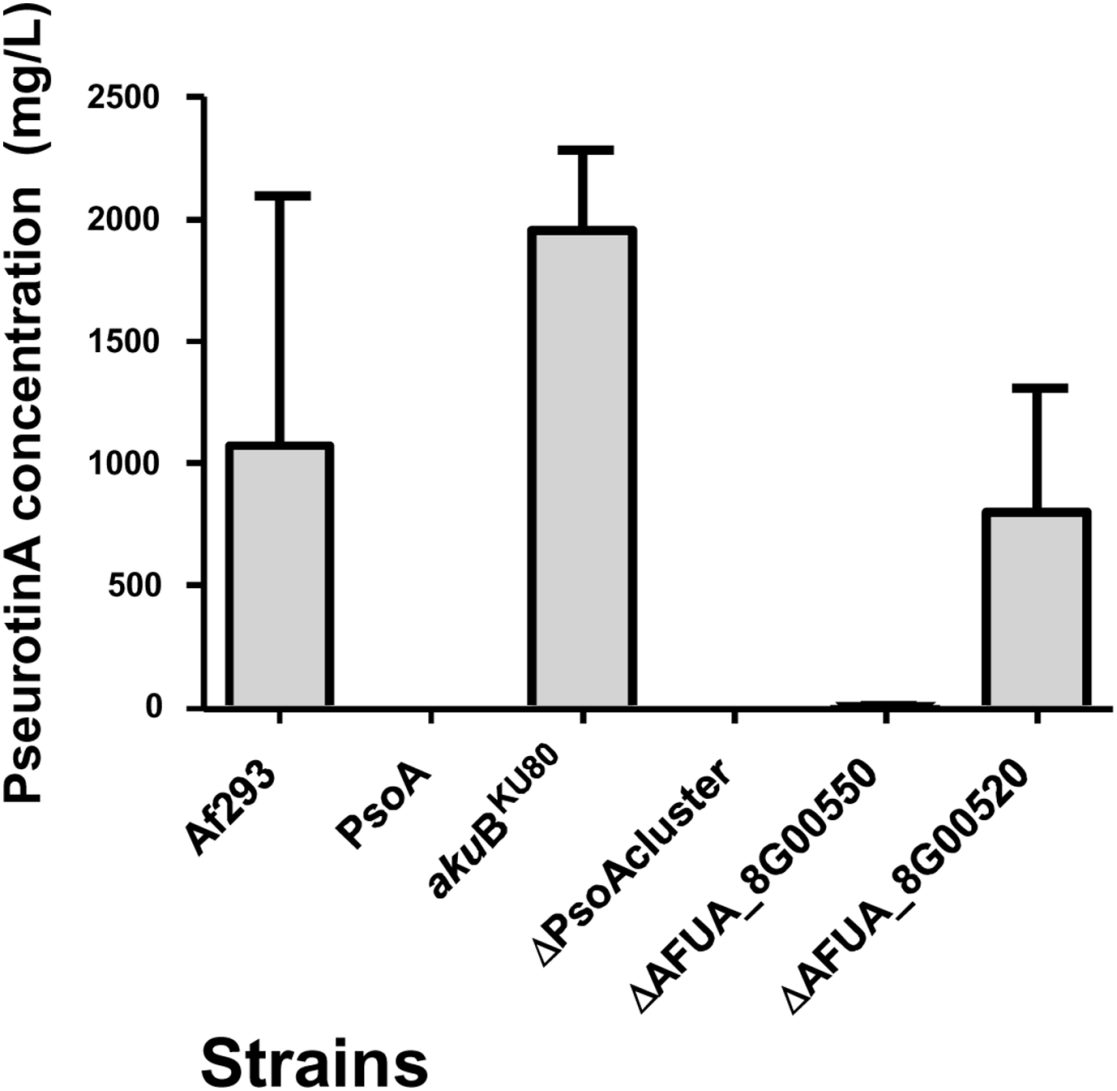
Quantification of pseurotin A by UPLC-ESI coupled to mass spectrometry in wild type (*A. fumigatus* CEA17_Δ*akuB* ^KU80^ referred as *akuB* ^KU80^) and mutant strains.

In our study phenotypic analyses of the newly constructed mutants was performed in parallel with a previously constructed *ΔpsoA* strain [47] which lacks the hybrid non-ribosomal-polyketide synthase PsoA (AFUA_8G00540), and its progenitor the Af293 parental strain (Table 1). Radial growth analyses revealed no growth defects amongst the PsoA cluster mutants (data not shown). Analysis of pseurotin production revealed that pseurotin biosynthesis was completely abrogated in mutants lacking the biosynthetic gene cluster (Figure 4) and further, that integrity of AFUA_8G00550 is required for pseurotin biosynthesis. Mass spectrometry conditions for Pseurotin A and internal standards are listed in Table 5. This result agrees with a recent study in which a role for the AFUA_8G00550 gene product in O-methylation of an intermediate metabolite has been demonstrated, and where elimination of this methylating activity limited the synthesis of pseurotin A [53]. In contrast, deletion of the AFUA_8G00520 gene did not eliminate pseurotin A biosynthesis compared to a congenic parental isolate.

**Table 5:** Mass spectrometry conditions for Pseurotin A and internal standards.

Type	Compound	Retention time(min)	Transitions	Conevoltage	Collisionenergy
Target	Pseurotin A	3.95	**432>316**	**30**	**10**
			432>348	15	10
Internal Standard (1)	3-F Pseurotin A	3.59	450>334	15	5
			450>366	15	5
Internal Standard (2)	2-F Pseurotin A	4.63	450>344	15	5
			450>366	15	5

*In bold is the quantification transition.

### Macrophage-mediated phagocytosis of *A. fumigatus,* and host cell damage

**Figure 5:**
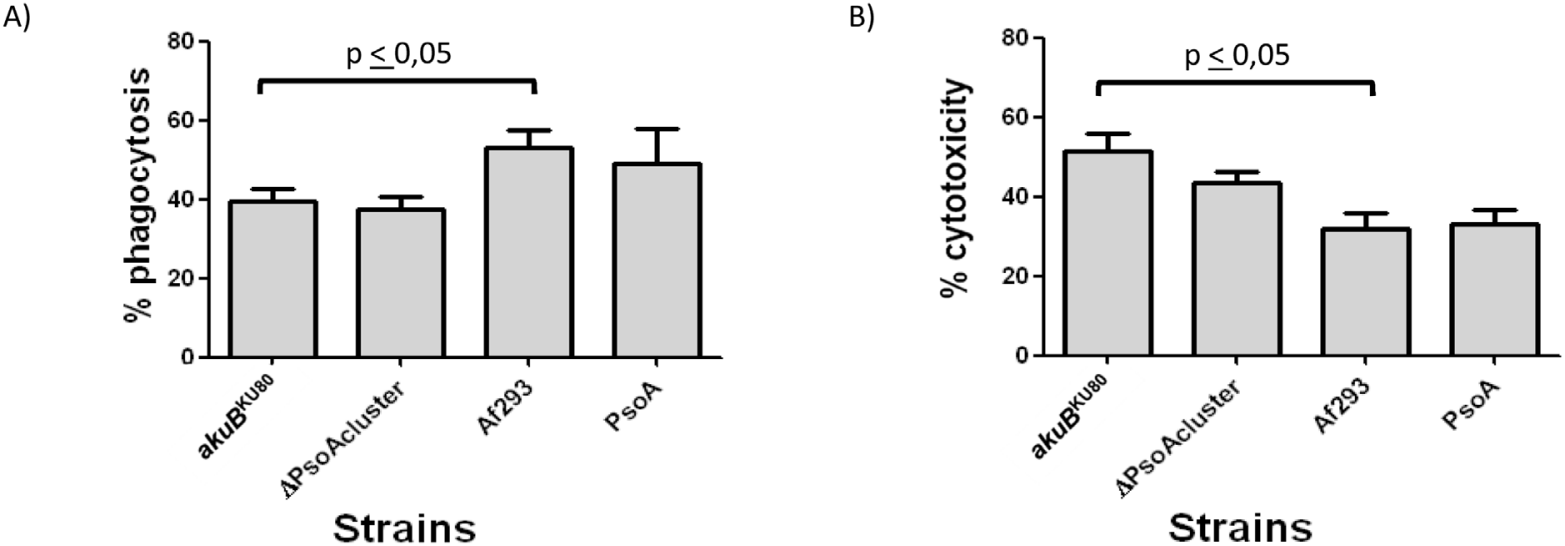
Effect of pseurotin A production upon interaction of *A. fumigatus* strains with mammalian cells. A) Percentage conidial phagocytosis following 2 h incubation with murine macrophages (RAW 264.7). B) Relative cytotoxicity (LDH release) after 24 h of co-incubation of *A. fumigatus* and human alveolar epithelial cells (A549). The statistical significance was calculated by using a nonparametric Mann-Whitney t test. A p value<0.05 was considered significant. *A. fumigatus* CEA17_Δ*akuB* ^KU80^ referred as *akuB* ^KU80^.

Genes of the pseurotin A biosynthetic gene cluster are co-ordinately upregulated, relative to laboratory culture, during initiation of murine infection [9]. This observation, coupled with reported activity of pseurotin A as an inducer of nerve-cell differentiation [48] and suppressor of immunoglobulin E production [49] prompted us to determine whether the absence of pseurotin A had any impact on the mammalian host response to *A. fumigatus* challenge. Since macrophages and lung epithelial cells constitute the main initial immunological barriers to *A. fumigatus* infection [56] the role of pseurotin A as a cytotoxic molecule was tested in these two cell types. Murine macrophages (RAW 264.7) and human lung epithelial cells (A549) were co-incubated with spores from either CEA17_Δ*akuB* ^KU80^ or pseurotin-deficient strains. For analysis of phagocytosis by macrophages, the proportion of unphagocytosed *A. fumigatus* spores was calculated, after two hours of host and pathogen co-incubation (Figure 5A). This analysis revealed no differences between pseurotin A-producing and non-producing isolates in either of the *A. fumigatus* CEA17_Δ*akuB* ^KU80^ or Af293 genetic backgrounds, however a significant difference in phagocytosis of isolates derived from CEA17_Δ*akuB* ^KU80^ and Af293 genetic backgrounds was discernable (Figure 5A). Relative cytotoxicity, to A549 epithelial cells, of pseurotin A-producing and non-producing isolates *A. fumigatus* was determined by release of LDH (Figure 5B). Again, no significant impact of pseurotin A upon epithelial cell lysis was measurable in our assays; however, we observed that *A. fumigatus* CEA17_Δ*akuB* ^KU80^ isolate was reproducibly more resistant than Af293 to phagocytosis by macrophages, and more cytotoxic to epithelial cells (Figure 5B).

### Screening of pathogenicity of *A. fumigatus* strains in *G. mellonella*

In keeping with the diverse bioactivity of fungal secondary metabolites, nematocidal properties of pseurotin A have previously been reported [57]. A precedent for the biosynthetic products of *A. fumigatus* gene clusters to moderate the outcome of host-pathogen interactions has recently been demonstrated, where deletion of the NRP synthetase Pes3 (AFUA_5G12730) significantly increases the virulence of *A. fumigatus* in wax moth larvae [58]. To test the role of pseurotin A in pathogenicity towards invertebrate hosts we utilized an established *G. mellonella* wax moth infection assay. This model has emerged as a useful alternative to mammalian infection assays for the study of fungal virulence and pathogenesis, fungus-host interactions, and antifungal drug efficacy [42], [59].

**Figure 6:**
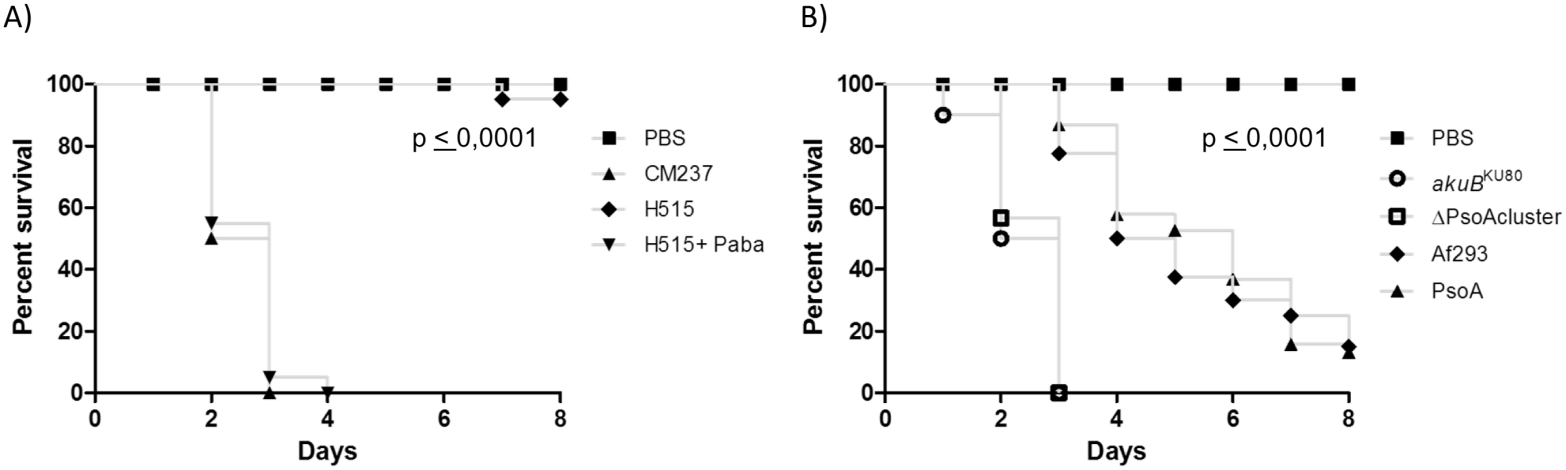
Survival of *G. mellonella* infected with *A. fumigatus*. A) Survival following infection with CM237 or H515, +4 µg/ml PABA. P value corresponds to comparison of survival rate between CM237 and H515 infected larvae. B) Survival following infection with wild type (CEA17_Δ*akuB* ^KU80^ (*akuB* ^KU80^) and Af293) or mutant strains (PsoA and ΔPsoAcluster); P value corresponds to comparison of survival rate between CEA17_Δ*akuB* ^KU80^ and Af293. A p value<0.01 was considered significant.

All *A. fumigatus* strains, with the exception of the H515 PABA auxotroph (Figure 6A) were able to kill the larvae at 37°C following injection of conidia into the larval haemocoel (Figure 6). In keeping with observations in murine models of infection, the H515 PABA auxotroph became pathogenic when administered in combination with exogenous PABA. Relative to the CEA17_Δ*akuB* ^KU80^ strain or Af293 strain, no significant differences in larval mortality were found following injection of pseurotin non-producing isolates (Figure 6B). Our data suggest that the pseurotin A gene cluster is not required for *A. fumigatus* virulence in *G. mellonella*. A full analysis in the mammalian host (under way in our laboratory) will be required to dismiss the involvement of this secondary metabolite in mammalian virulence.

We observed differential pathogenicity traits in Af293 and CEA17_Δ*akuB* ^KU80^ which extend to pathogenicity in invertebrate hosts (Figure 6B). Such strain-dependent variance of the host response to *A. fumigatus* has been also recently reported for CEA10 (which is the CEA17_Δ*akuB* ^KU80^ progenitor) which elicits a stronger inflammatory response, based on the cytokine secretion profile of *Aspergillus*-stimulated dentritic cells, compared to Af293 [60].

### Conclusions

We have developed a new protocol based on *E. coli* recombineering methodology to efficiently target genes and gene clusters in *A. fumigatus*. Advantages of this system.

include: (i) a single PCR is required for construction of gene replacement cassettes; (ii) maximisation of flanking regions promotes efficient sequence replacement in *A. fumigatus;* (iii) the approach works well in wild-type clinical isolates. Our methodology significantly expands the toolkit available for manipulation of *A. fumigatus* gene clusters.

## Supporting Information

Figure S1 A) Schematic representation of gene AFUA_1G17640 (*reg*A) replacement by BSM-Z/P cassette in *A. fumigatus* CEA17_Δ*akuB* ^KU80^. (B) Schematic representation of the *reg*A region following a homologous recombination event with the BAC CC_e12.9 disruption cassette. A NotI restriction site is introduced by the BSM-Z/P cassette. (C) Southern blot analyses of CEA17_Δ*akuB* ^KU80^ (lane 1) and *Δreg*A transformant (lane 2) gDNA which was digested with SacI. Blots were probed with a 600 bp fragment of the zeocin cassette. *reg*A deletion is indicated by a single SacI fragment of 12.5 Kb observed for *Δreg*A gDNA. (D) Gel electrophoresis image of diagnostic PCR to confirm *reg*A gene deletion. Primers 5′_640 and 3′_640 were used to amplify the *reg*A region from CEA17_Δ*akuB* ^KU80^ and putative *Δreg*A transformant gDNA. PCR amplicons from CEA17_Δ*akuB* ^KU80^ and *Δreg*A were NotI digested and analysed using gel electrophoresis, which demonstrated wild-type banding patterns in the CEA17_Δ*akuB* ^KU80^ (lane 1) and introduction of the NotI site by the zeocin cassette in the transformant strain (lane 2). PCR using primers internal to the *reg*A coding sequence demonstrated a product of the expected 1.55 kb size from CEA17_Δ*akuB* ^KU80^ gDNA template (lane 3) but no product from *Δreg*A transformant (lane 4), indicating *Δreg*A gene replacement.
(TIF)

Figure S2 A) Schematic view of AFUA_8G00520 replacement by BSM-A/H cassette in *A. fumigatus* CEA17_Δ*akuB* ^KU80^. B and C) Southern blot analysis of AFUA_8G00520 deleted mutant and wild type (WT) strains. Expected hybridization band pattern: (1) 5042 bp for WT, and (2, 3, 4) 6724 bp for the mutants.
(TIF)

Figure S3 A) Schematic view of AFUA_8G00550 replacement by BSM-A/H cassette in *A. fumigatus* CEA17_Δ*akuB* ^KU80^. B and C) Southern blot analysis of AFUA_8G00550 deleted mutant and wild type (WT) strains. Expected hybridization band pattern: (1, 2, 3) 3772 bp for the mutants and (4) 756 bp for WT.
(TIF)

Table S1 BAC library.
(XLS)

File S1 BAC clones sequences.
(GZ) Click here for additional data file.

Protocol S1 Protocol for recombineering.
(DOCX)
