# Supplementary figures and images for: A Modified Recombineering Protocol for the Genetic Manipulation of Gene Clusters in Aspergillus fumigatus
Source: PLoS One. 2014 Nov 5;9(11):e111875. doi: 10.1371/journal.pone.0111875 (PMC4221250; doi:10.1371/journal.pone.0111875)

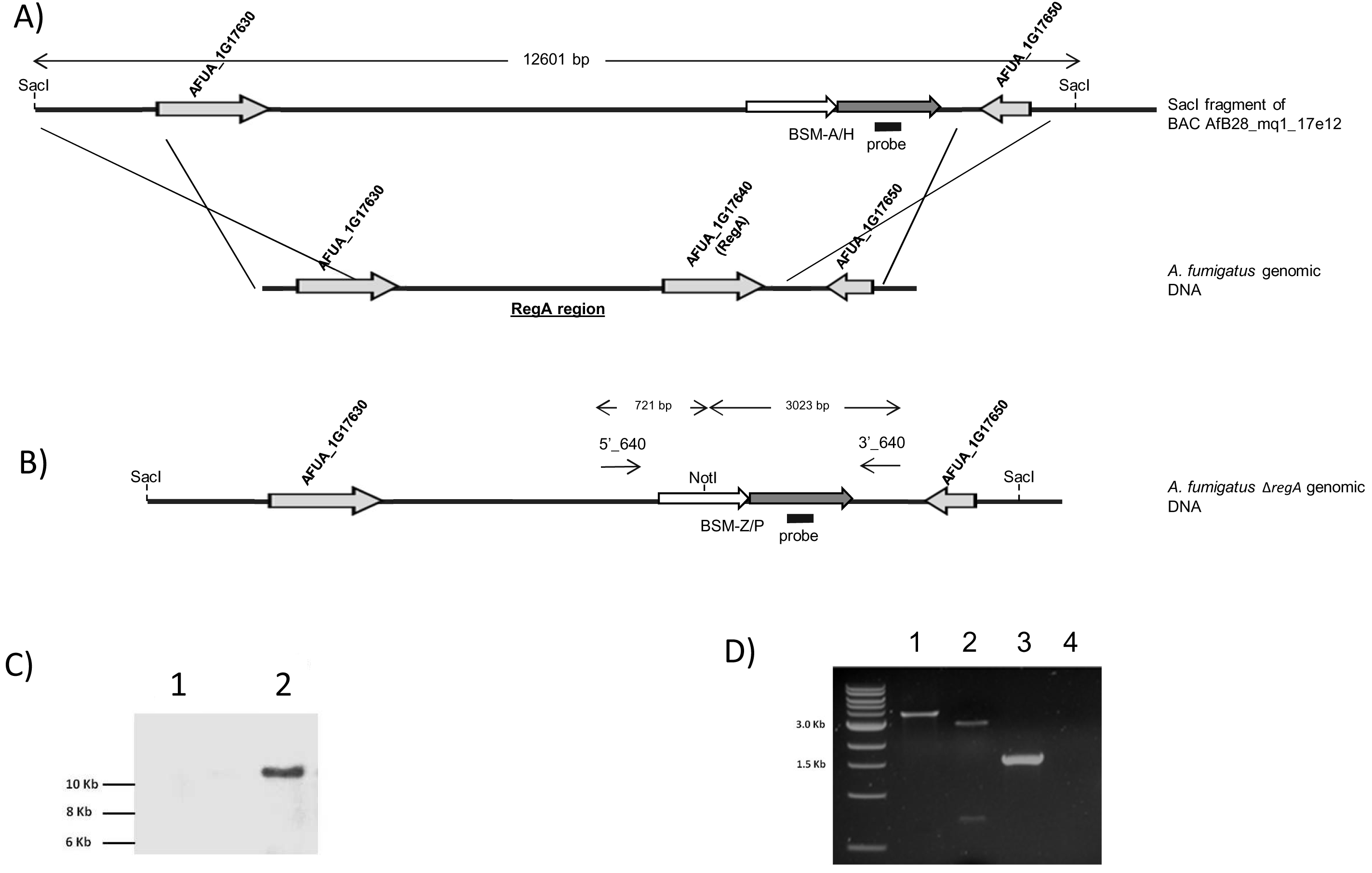

Supplement: Figure S1 — A) Schematic representation of gene AFUA_1G17640 (regA) replacement by BSM-Z/P cassette in A. fumigatus CEA17_ΔakuB KU80. (B) Schematic representation of the regA region following a homologous recombination event with the BAC CC_e12.9 disruption cassette. A NotI restriction site is introduced by the BSM-Z/P cassette. (C) Southern blot analyses of CEA17_ΔakuB KU80 (lane 1) and ΔregA transformant (lane 2) gDNA which was digested with SacI. Blots were probed with a 600 bp fragment of the zeocin cassette. regA deletion is indicated by a single SacI fragment of 12.5 Kb observed for ΔregA gDNA. (D) Gel electrophoresis image of diagnostic PCR to confirm regA gene deletion. Primers 5′_640 and 3′_640 were used to amplify the regA region from CEA17_ΔakuB KU80 and putative ΔregA transformant gDNA. PCR amplicons from CEA17_ΔakuB KU80 and ΔregA were NotI digested and analysed using gel electrophoresis, which demonstrated wild-type banding patterns in the CEA17_ΔakuB KU80 (lane 1) and introduction of the NotI site by the zeocin cassette in the transformant strain (lane 2). PCR using primers internal to the regA coding sequence demonstrated a product of the expected 1.55 kb size from CEA17_ΔakuB KU80 gDNA template (lane 3) but no product from ΔregA transformant (lane 4), indicating ΔregA gene replacement. (TIF) [file pone.0111875.s001.tif]

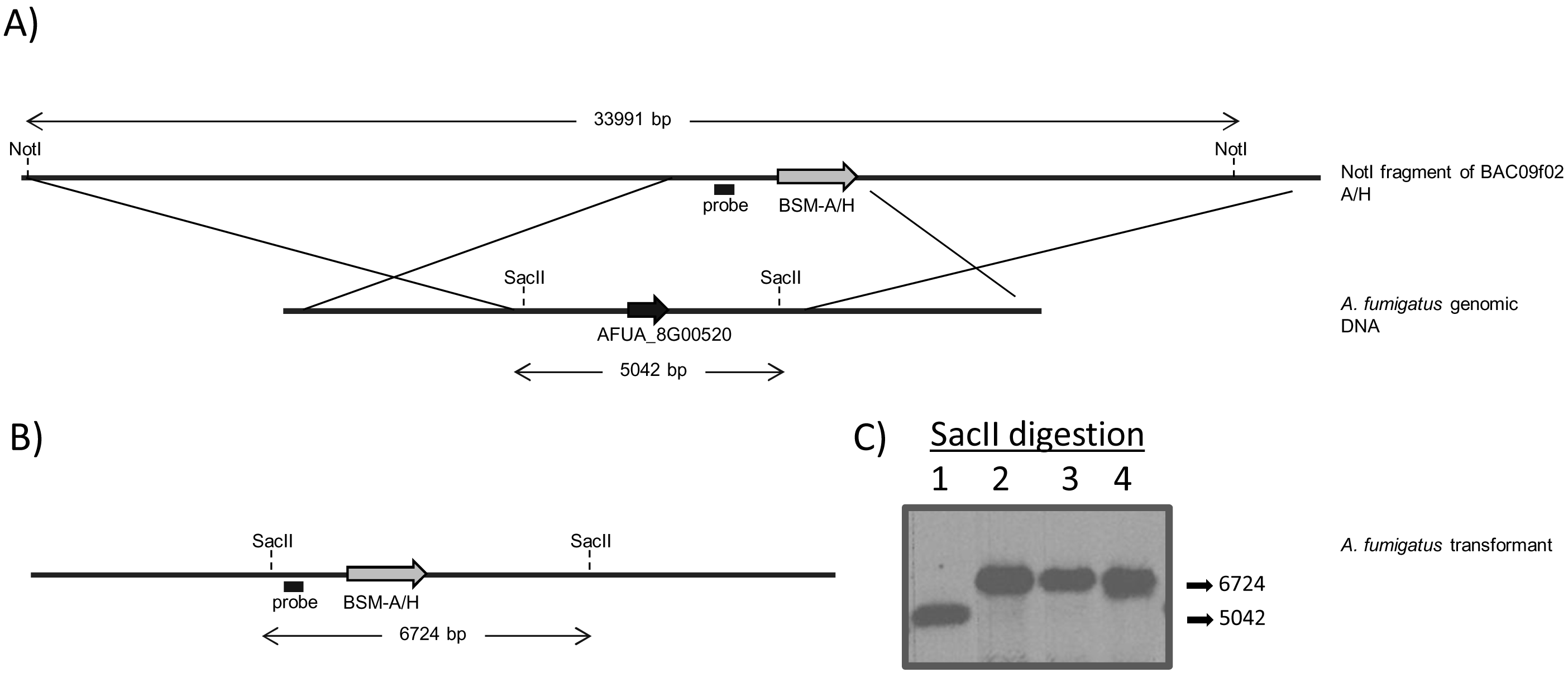

Supplement: Figure S2 — A) Schematic view of AFUA_8G00520 replacement by BSM-A/H cassette in A. fumigatus CEA17_ΔakuB KU80. B and C) Southern blot analysis of AFUA_8G00520 deleted mutant and wild type (WT) strains. Expected hybridization band pattern: (1) 5042 bp for WT, and (2, 3, 4) 6724 bp for the mutants. (TIF) [file pone.0111875.s002.tif]

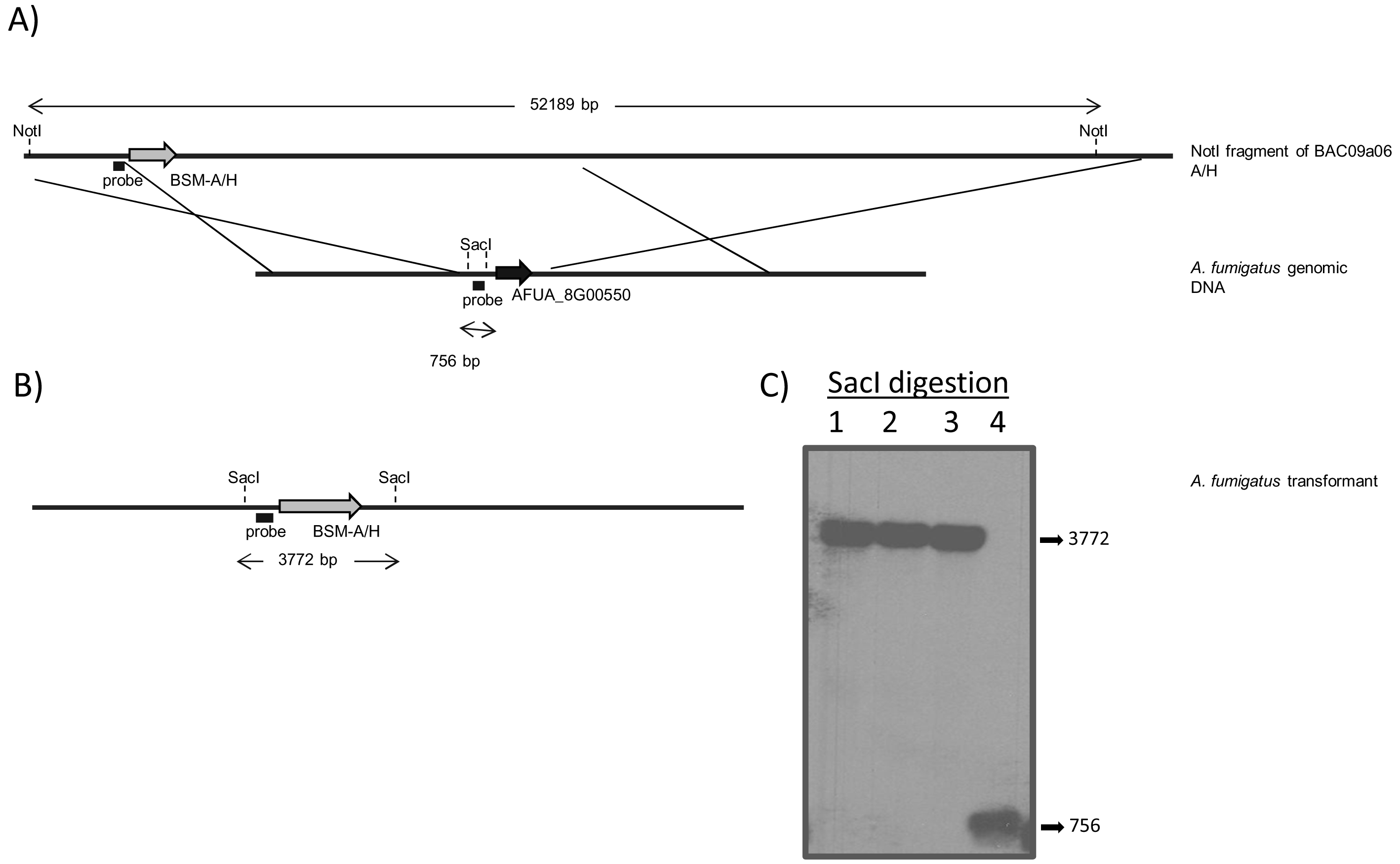

Supplement: Figure S3 — A) Schematic view of AFUA_8G00550 replacement by BSM-A/H cassette in A. fumigatus CEA17_ΔakuB KU80. B and C) Southern blot analysis of AFUA_8G00550 deleted mutant and wild type (WT) strains. Expected hybridization band pattern: (1, 2, 3) 3772 bp for the mutants and (4) 756 bp for WT. (TIF) [file pone.0111875.s003.tif]
