# Supplementary material for: A Modified Recombineering Protocol for the Genetic Manipulation of Gene Clusters in Aspergillus fumigatus
Source: PLoS One. 2014 Nov 5;9(11):e111875. doi: 10.1371/journal.pone.0111875 (PMC4221250; doi:10.1371/journal.pone.0111875)
Supplement: Protocol S1 — Protocol for recombineering. (DOCX) [file pone.0111875.s006.docx]

**Protocol S1**

Protocol for recombineering, based on [27]:

BAC clone infection with replication deficient λ phage:

1. Inoculate cells into 5 ml LB (with Chloramphenical (12.5 µg/ml), for overnight growth at 37°C at 180 rpm.
2. Split the cells into 1ml aliquots
3. Cells are collected by centrifugation at 10,000xg and washed once with 1 ml 10 mM MgSO_4_
4. The pellet is resuspended in 100 µl 10 mM MgSO_4_
5. Add 1 μl containing greater than 1 million lambda phage to 100 µl of the BAC cells
6. Incubate the mixture at 32°C, 180 rpm for 20 min.
7. Add 1 ml of LB to the tube and incubate for 1 hour at 32°C, 180rpm.
8. Plate 100 ul on LB tetracycline (12.5 ug/ml) plates and incubate overnight at 32°C.

BAC Recombineering:

1. Pick up one TetR colony and inoculate into 1ml LB with chloramphenicol (12.5 µg/ml) and tetracycline (12.5 ug/ml each) grow them overnight growth at 32^o^C, with shaking at 180 rpm
2. Inoculate 1 ml of fresh LB with 25, 35, 45 and 55 μl of each overnight culture
3. Incubate for 2 h at 32^o^C, 180 rpm (OD must be (OD_540_~0.5-0.6))
4. Transfer to eppendorfs
5. Incubate 15 min at 42^o^C in a water bath
6. Very quickly transfer for 2 min on ice
7. Collect the cells by centrifugation at 10,000xg and combine them Wash with 1 ml of 100 mM MgCl_2_ / 100 mM CaCl_2_ solution.
8. Wash with 1 ml 100 mM CaCl_2_
9. Wash with 1 ml 100 mM CaCl_2_
10. Resuspend each tube with 50 μl CaCl_2_ containing the PCR product (replacement cassette)
11. Incubate for 20 min on ice
12. Incubate for 2 min at 42ºC
13. Incubate for 2 min on ice
14. Add 1 ml LB and incubate for at least 1h at 32^o^C
15. Plate on LB plates plus appropriate selection* and incubate at 32^o^C O/N

*Antibiotics and concentrations used for recombineering:

| Ampicillin | 100 ug/ml | Dissolved in water, filtered |
| --- | --- | --- |
| Chloramphenicol | 10 or 12.5 ug/ml | 100 % in ethanol |
| Tetracycline | 12.5 ug/ml | 50% water/ethanol filtered |
| Zeocin | 25 ug/ml | Dissolved in water, filtered |
